# Supplementary material for: A molecular map of the human spinal dorsal and ventral horn defines arrangement of neuronal types and glial sex differences
Source: bioRxiv. 2025 Nov 1:2025.10.31.685953. Preprint. [Version 1] doi: 10.1101/2025.10.31.685953 (PMC12636303; doi:10.1101/2025.10.31.685953)
Supplement: Supplement 3 [file media-3.pdf]

Donor #	Age	Sex	COD	# Cells		Median reads per nuclei		Median genes per nuclei		Median UMI counts per nuclei		Total genes detected	
				DH	VH	DH	VH	DH	VH	DH	VH	DH	VH
1	23	Female	Anoxia	6,179	6,845	6,448	6,775	2,009	2,069	3,207	3,380	16,897	16,736
2	20	Male	GSW	4,935	7,729	7,637	7,166	2,299	2,220	3,813	3,568	17,451	17,159
3	32	Male	GSW	5,401	5,160	6,870	6,697	2,164	2,136	3,405	3,327	16,960	16,489
4	36	Female	Anoxia OD	6,225	6,992	5,829	4,824	1,872	1,574	2,896	2,399	16,744	16,576
5	43	Male	MVA	4,173	6,207	7,573	5,551	2,288	1,801	3,784	2,765	16,743	16,582
6	46	Female	MVA	5,594	5,734	6,368	6,098	1,977	1,900	3,179	3,027	17,041	16,658
7	50	Female	Anoxia	7,419	3,340	8,051	8,117	2,429	2,427	4,010	4,039	16,885	16,619
8	56	Male	Anoxia	5,653	5,307	5,138	6,446	1,746	2,079	2,548	3,198	17,176	17,169
9	39	Male	Anoxia OD	12,810	4,970	7,814	11,975	1,954	2,722	3,211	4,878	17,788	17,804
10	37	Female	GSW	14,319	2,341	13,967	17,409	3,285	3,506	5,707	7,100	17,720	16,688
11	60	Female	Anoxia	6,816	7,914	14,512	11,550	3,260	2,635	5,930	4,725	17,780	17,466

Supplemental Table 1. Quality control metrics per sample across all 11 donors divided by dorsal horn and ventral horns.

(Attached as separate excel file)

Supplemental Table 2. A list of genes included on the custom 480 gene 10X Xenium panel.

(Attached as separate excel file)

Supplemental Table 3. Pseudobulk Expression of Variable Cell Surface Genes in Mouse and Human Lumbar Spinal Cord

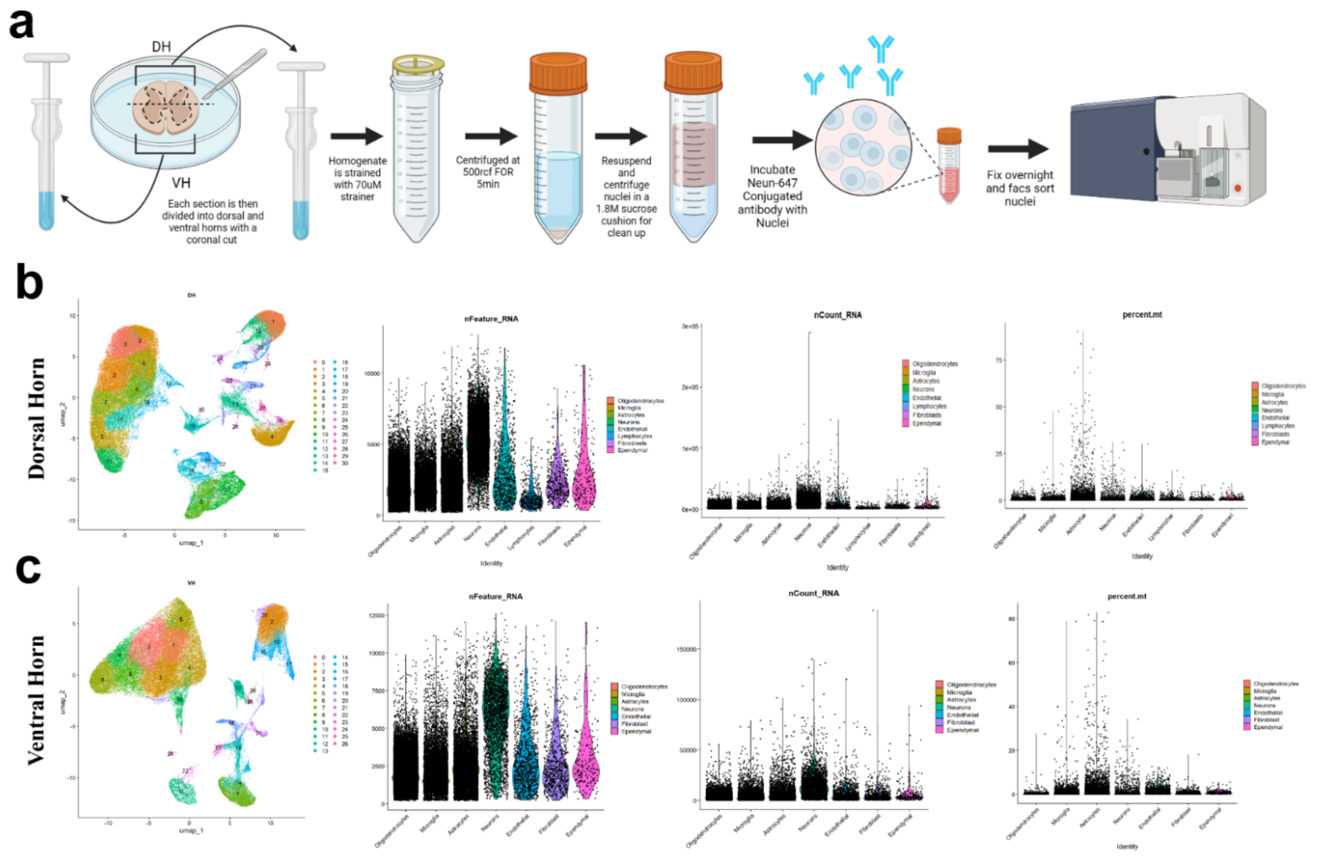

Supplemental Figure 1. a. Workflow of single nuclei sequencing extraction in the human spinal cord using FACS sorting. b. UMAP showing 31 clusters of the dorsal horn and the violin plots for three quality control metrics across all cells demonstrating high number of detected features, a good number of RNA molecules, and low percentage of mitochondrial gene expressions. c. UMAP showing 30 clusters of the ventral horn and the violin plots for three quality control metrics across all cells demonstrating high number of detected features, the RNA molecules, and low percentage of mitochondrial gene expressions.

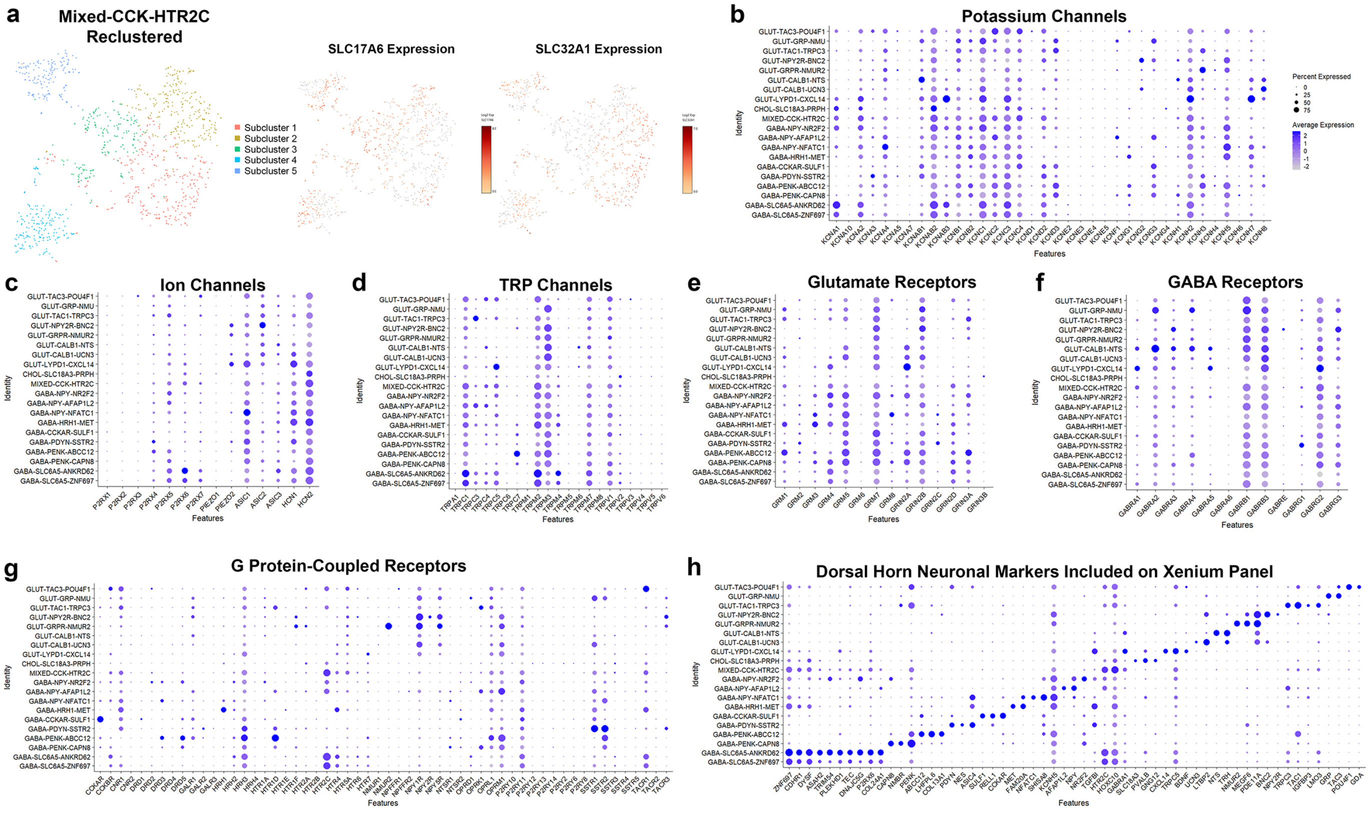

Supplemental Figure 2. a, Subclustering the mixed dorsal horn population of neurons (MIXED-CCK-HTR2C) revealed five transcriptomically similar clusters, all containing excitatory (SLC17A6+) and inhibitory (SLC32A1+) nuclei. Dot plots showing expression of potassium channel components (b), ion channels including purinurgic and ASIC channels (c), TRP channels (d), glutamate receptor subunits (e), GABA receptor subunits (f), G protein-coupled receptors (g) and marker genes used to identify each population on the Xenium panel (h) in dorsal horn neuronal clusters.

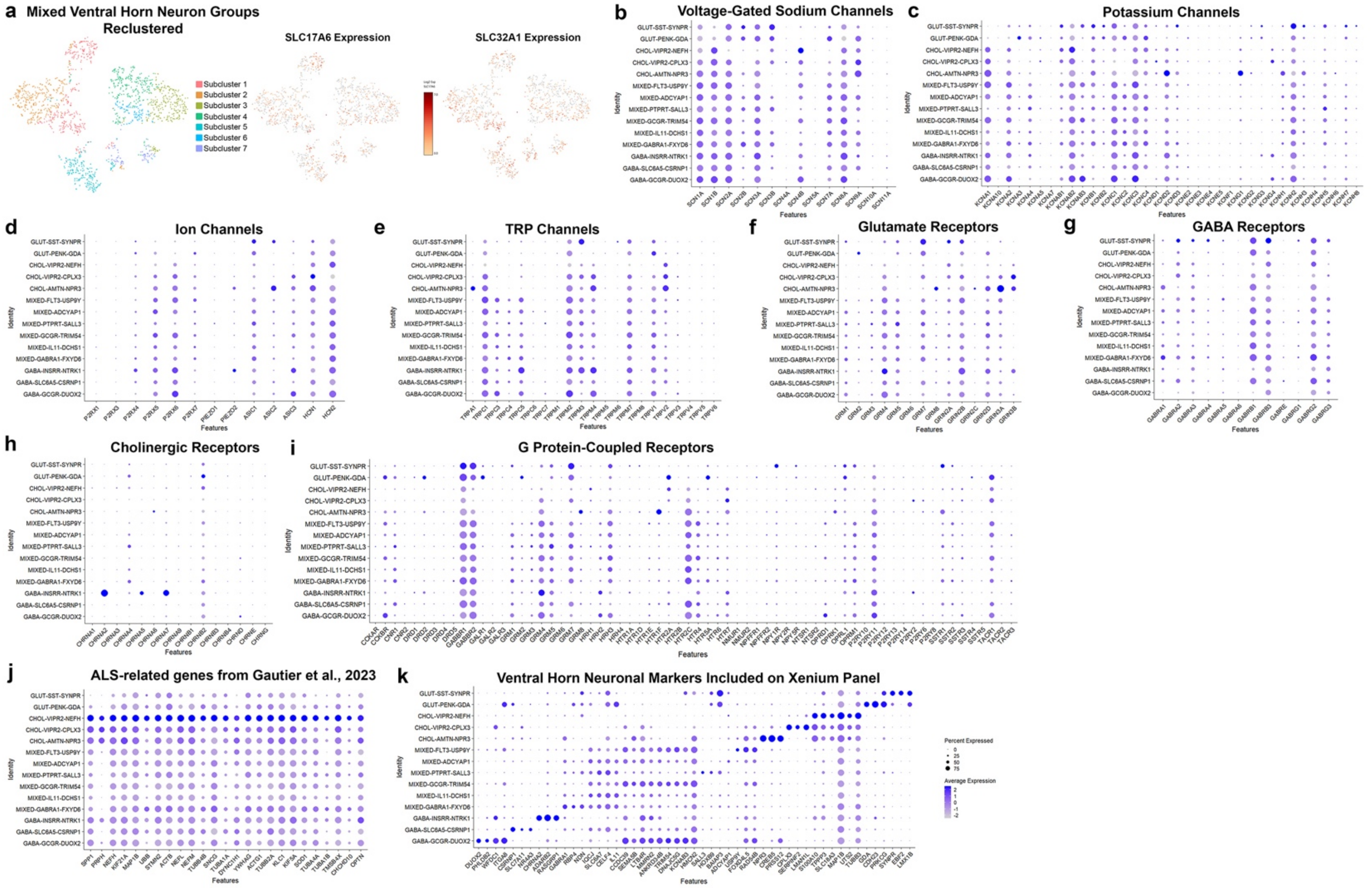

Supplemental Figure 3. a, Subclustering the six mixed ventral horn populations of neurons produced seven transcriptomically similar clusters, all containing excitatory (SLC17A6+) and inhibitory (SLC32A1+) nuclei. Dot plots showing expression of voltage-gated sodium channels (b), potassium channel components (c), ion channels including purinurgic and ASIC channels (d), TRP channels (e), glutamate receptor subunits (f), GABA receptor subunits (g), cholinergic receptors (h), G protein-coupled receptors (i), ALS-related genes from Gautier et al., (2023; j) and marker genes used to identify each population on the Xenium panel (k) in ventral horn neuronal clusters.

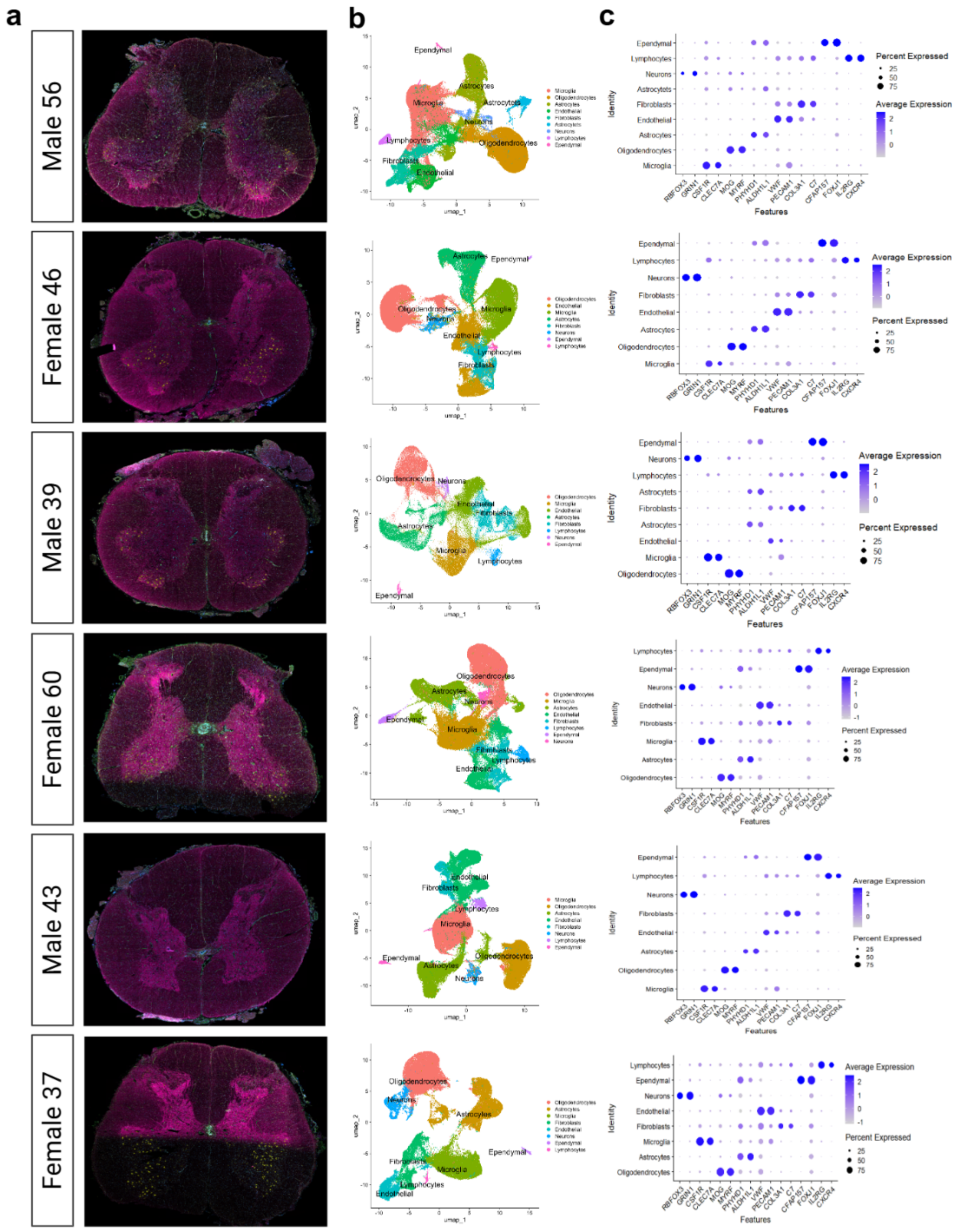

Supplemental Figure 4. Xenium spatial transcriptomics on each individual sample. a. Initial cell segmentation output from Xenium using Xenium explorer across six samples. b. UMAP plots showing named clusters for each of the six individual samples. c. Dot plot displaying the marker genes used to annotate clusters across the six samples.

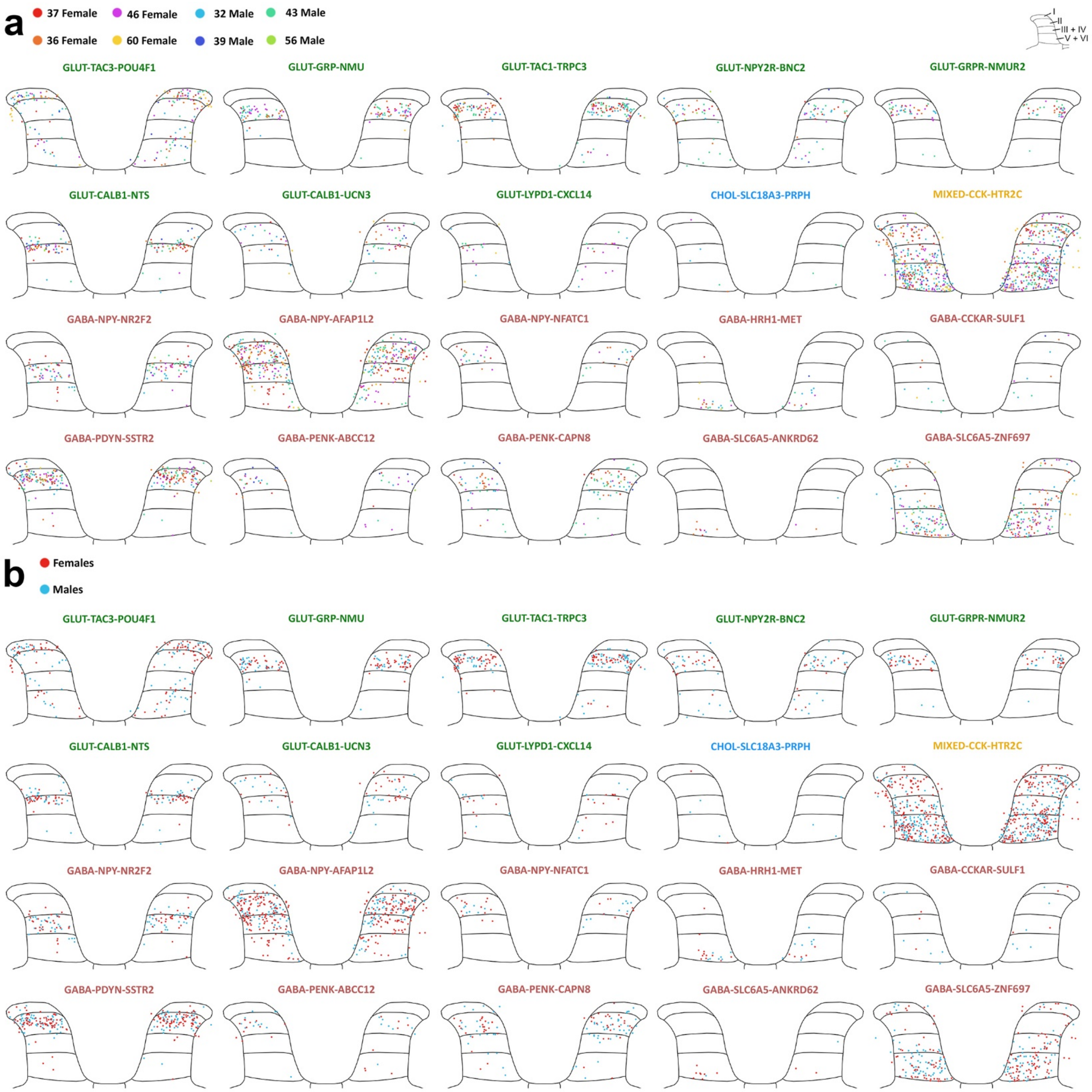

Supplemental Figure 5. Spatial organization of dorsal horn clusters shown per individual donor and grouped by sex.

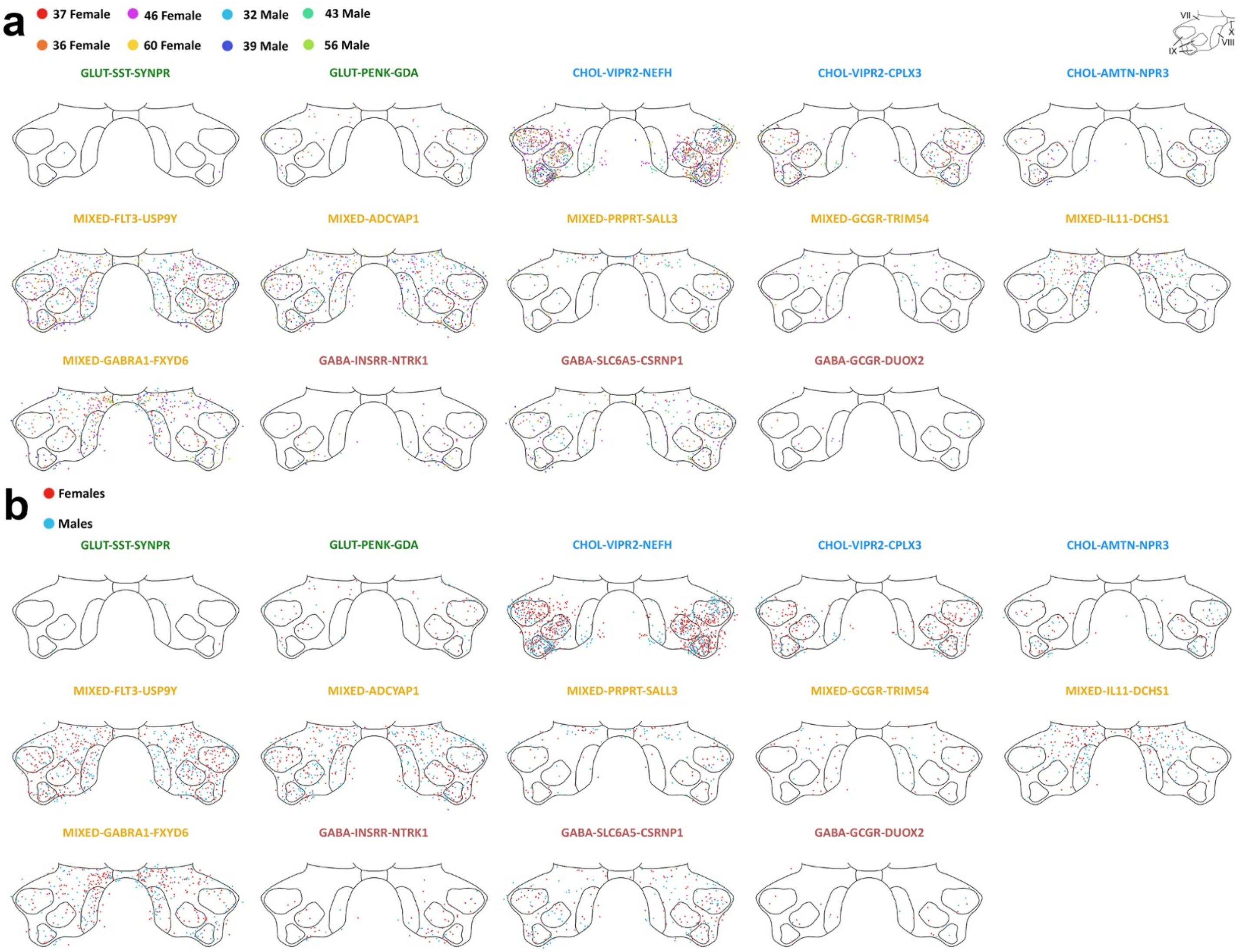

Supplemental Figure 6. Spatial organization of ventral horn clusters shown per individual donor and grouped by sex.

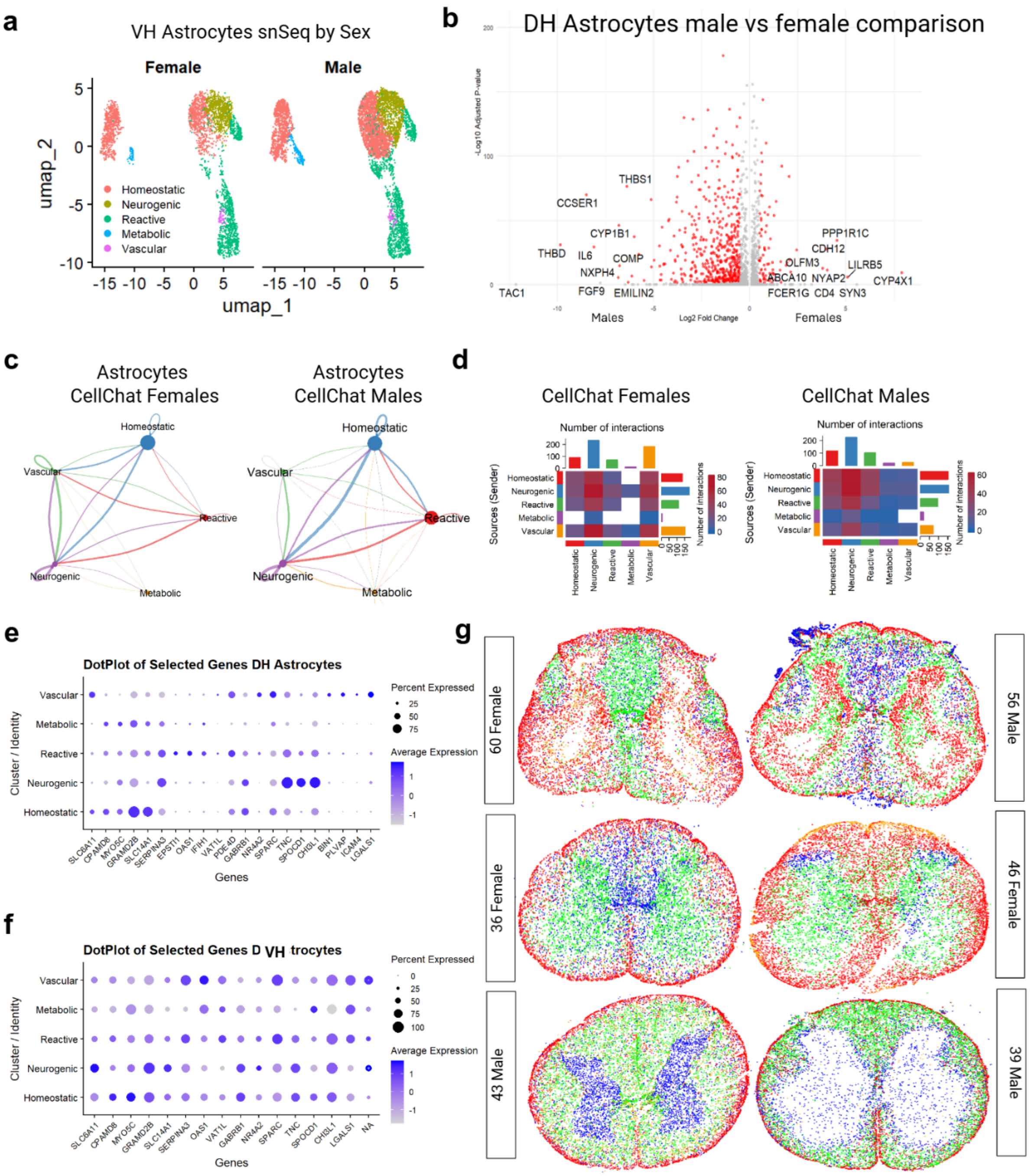

Supplemental Figure 7. a. UMAP showing subclustered ventral horn astrocytes split by sex. b. volcano plot demonstrating differential gene expression changes between males and female in dorsal horn astrocytes. c. Intercellular communication network reflecting the numbers and intensity of interactions between the functional categories in the dorsal horn astrocytes split by males and females. d. Representative heatmap of the intercellular communication network between astrocytes in males and females. e Dot plots demonstrating genes of interest for categorizing astrocytes by function in the dorsal horn. f. Dot plots of categorizing genes in the ventral horn. g. Spatial organization of astrocytes categorized by function across 6 remaining donors.

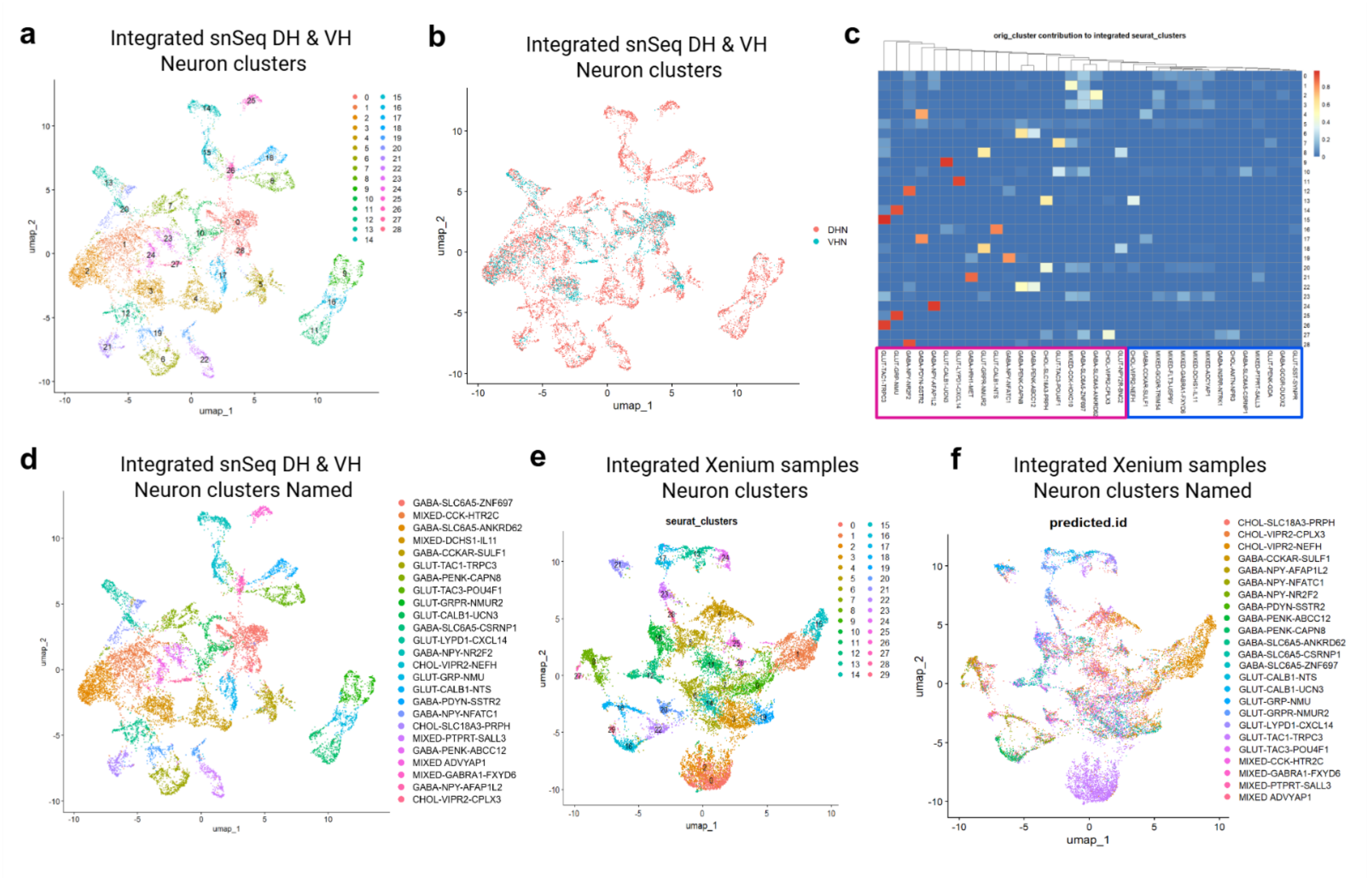

Supplemental Figure 8. a. UMAP of integrated ventral horn and dorsal horn neuronal clusters yielding 29 clusters. b. UMAP of integrated neuronal clusters showing distribution of ventral horn (blue) and dorsal horn (red) nuclei. c. heatmap to visualize the outcome of label transfer, where predicted cell type annotations from the dorsal horn and ventral horn neuronal clusters were mapped onto the clusters of the integrated neuronal object. d. UMAP depicting the final cluster assignment of the integrated single nuclei sequencing neuronal clusters. e. UMAP of integrated xenium neuronal clusters. f. UMAP of final cluster assignment of the integrated xenium neuronal clusters using label transfer.

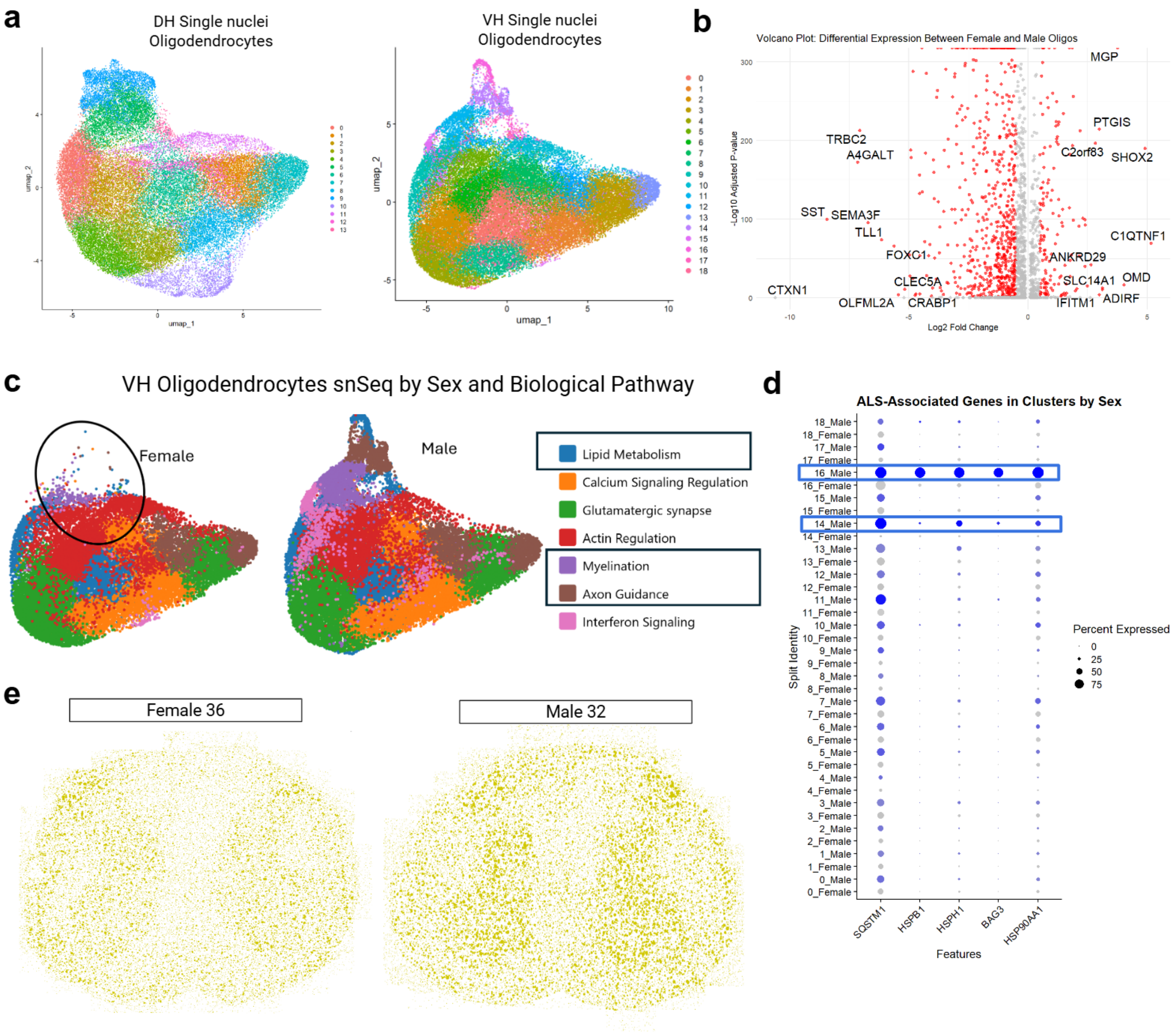

Supplemental Figure 9: a. single nuclei sequencing UMAPs of oligodendrocytes from the dorsal horn (14 clusters) and ventral horn (19 clusters). b. Volcano plot demonstrating differential gene expression changes between males (left) and female (right) in dorsal horn astrocytes. c. single nuclei sequencing UMAPs of oligodendrocytes split by sex and categorized by biological pathway. d. Dot plot looking at ALS associated genes that are highly expressed in clusters that are male exclusive (14 & 16). e. Xenium spatial distribution of oligodendrocytes in two representative male and female donors.

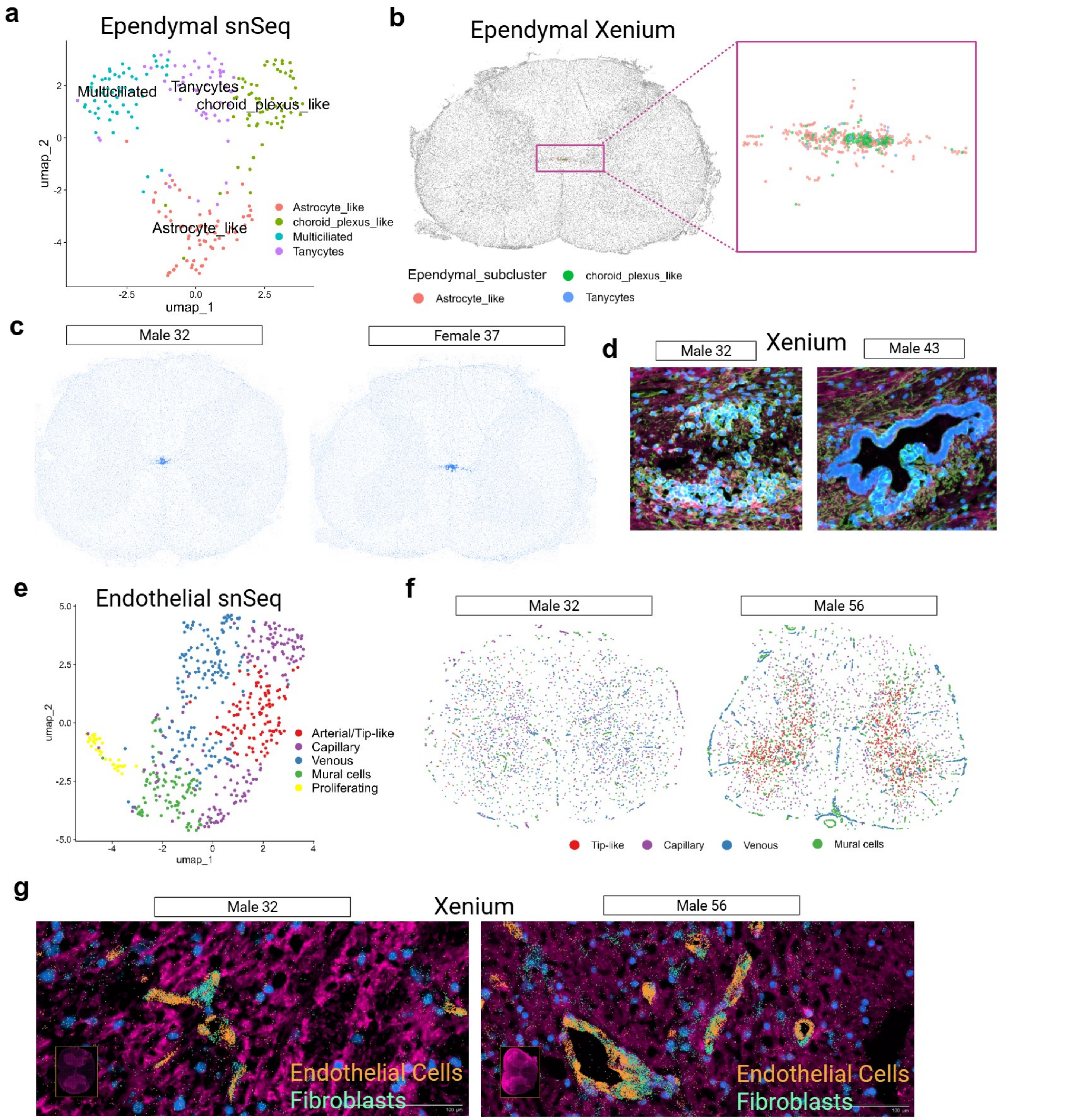

Supplemental Figure 10: **a**. single nuclei sequencing UMAP of subclustered endothelial cell categories. **b**. Spatial distribution of endothelial cells in one xenium sample. **c**. Representative male and female samples of endothelial cell in xenium. **d**. Xenium image of differences in stenosis of central canal between two donors demonstrating it is age independent. e. single nuclei sequencing UMAP of subclustered endothelial cell categories. **f**. Xenium spatial distribution of endothelial cells between a

younger and an older male sample. **g**. Xenium image of differences in size of vascular formations across two different aged samples in the dorsal horn.
